# Assessment of Discordance Between Surrogate Care Goals and Medical Treatment Provided to Older Adults With Serious Illness

**DOI:** 10.1001/jamanetworkopen.2020.5179

**Published:** 2020-05-19

**Authors:** Amber R. Comer, Susan E. Hickman, James E. Slaven, Patrick O. Monahan, Greg A. Sachs, Lucia D. Wocial, Emily S. Burke, Alexia M. Torke

**Affiliations:** 1 Department of Health Sciences, Indiana University School of Health and Human Sciences, Indianapolis; 2 Indiana University Center for Aging Research, Regenstrief Institute, Inc, Indianapolis; 3 Indiana University Purdue University Indianapolis Research in Palliative and End-of-Life Communication and Training (RESPECT) Center, School of Nursing, Indiana University, Indianapolis; 4 Department of Community and Health Systems, School of Nursing, Indiana University, Indianapolis, Indiana; 5 Fairbanks Center for Medical Ethics, Indiana University Health, Indianapolis; 6 Department of Biostatistics, Indiana University School of Medicine, Indianapolis, Indiana; 7 Division of General Internal Medicine and Geriatrics, Indiana University School of Medicine, Indianapolis

## Abstract

**Importance:**

An important aspect of high-quality care is ensuring that treatments are in alignment with patient or surrogate decision-maker goals. Treatment discordant with patient goals has been shown to increase medical costs and prolong end-of-life difficulties.

**Objectives:**

To evaluate discordance between surrogate decision-maker goals of care and medical orders and treatments provided to hospitalized, incapacitated older patients.

**Design, Setting, and Participants:**

This prospective cohort study included 363 patient-surrogate dyads. Patients were 65 years or older and faced at least 1 major medical decision in the medical and medical intensive care unit services in 3 tertiary care hospitals in an urban Midwestern area. Data were collected from April 27, 2012, through July 10, 2015, and analyzed from October 5, 2018, to December 5, 2019.

**Main Outcomes and Measures:**

Each surrogate’s preferred goal of care was determined via interview during initial hospitalization and 6 to 8 weeks after discharge. Surrogates were asked to select the goal of care for the patient from 3 options: comfort-focused care, life-sustaining treatment, or an intermediate option. To assess discordance, the preferred goal of care as determined by the surrogate was compared with data from medical record review outlining the medical treatment received during the target hospitalization.

**Results:**

A total of 363 dyads consisting of patients (223 women [61.4%]; mean [SD] age, 81.8 [8.3] years) and their surrogates (257 women [70.8%]; mean [SD] age, 58.3 [11.2] years) were included in the analysis. One hundred sixty-nine patients (46.6%) received at least 1 medical treatment discordant from their surrogate’s identified goals of care. The most common type of discordance involved full-code orders for patients with a goal of comfort (n = 41) or an intermediate option (n = 93). More frequent in-person contact between surrogate and patient (adjusted odds ratio [AOR], 0.43; 95% CI, 0.23-0.82), patient residence in an institution (AOR, 0.44; 95% CI, 0.23-0.82), and surrogate-rated quality of communication (AOR, 0.98; 95% CI, 0.96-0.99) were associated with lower discordance. Surrogate marital status (AOR for single vs married, 1.92; 95% CI, 1.01-3.66), number of family members involved in decisions (AOR for ≥2 vs 0-1, 1.84; 95% CI, 1.05-3.21), and religious affiliation (AOR for none vs any, 4.87; 95% CI, 1.12-21.09) were associated with higher discordance.

**Conclusions and Relevance:**

This study found that discordance between surrogate goals of care and medical treatments for hospitalized, incapacitated patients was common. Communication quality is a modifiable factor associated with discordance that may be an avenue for future interventions.

## Key Points

**Question:**

How frequent is discordance between surrogate decision-maker goals of care and medical treatments provided to hospitalized, incapacitated patients?

**Findings:**

In this prospective cohort study of 363 patient-surrogate dyads, 169 (46.6%) received at least 1 medical treatment discordant from their surrogate’s identified goals of care, including 10 patients who received cardiopulmonary resuscitation.

**Meaning:**

This study found many instances of patients receiving aggressive medical interventions discordant with the surrogate’s goal for medical care.

## Teaser

This cohort study assesses discordance between surrogate decision-maker goals of care and medical orders and treatments provided to hospitalized, incapacitated older patients.

## Introduction

High-quality care for seriously ill patients requires concordance among values, goals, and medical treatments.^1,2,3,4,5,6,7,8,9^ The American Academy of Hospice and Palliative Medicine uses concordance as a quality metric in evaluating care for seriously ill patients.^10^ Prior studies have evaluated various types of discordance, including patient perceptions that the plan of care was concordant with their wishes and concordance between clinicians’ and patients’ preferences, between patient and surrogate decision-maker preferences,^11,12,13,14^ and between advance care planning interventions and treatments received.^15,16^ Treatment discordant with the patient’s goals has been shown to increase medical costs and prolong end-of-life difficulties.^6,7,8,9,10,11^ In contrast, treatment that is concordant or aligned with the patient’s goals decreases anxiety, depression, trauma, and regret while improving trust, peacefulness, and satisfaction.^1,2,3,4,5,6,7,8,9,10,11^

For patients who lack decisional capacity, surrogate decision-makers are asked to provide consent for treatments based on the patient’s advance directives, goals, and values or best interests.^17^ If patient preferences have been previously documented, surrogate decisions can be compared with earlier patient preferences.^14^ However, in many cases, patient wishes for treatment are not well-documented, and surrogates are called on to make judgments about goals of care. Concordance between the surrogate’s judgment of the best goal of care and treatment received by the patient becomes a marker of quality. One study of surrogate preferences for patients with advanced dementia in a nursing home found that approximately 30% of family members believed their preferred plan of care was not the same as the preferences of the nursing home staff. Also, many patients whose family had a preferred plan of comfort lacked do-not-resuscitate (DNR) or other orders limiting life-sustaining treatment.^18^ Because of the potential for life-sustaining treatments in the hospital setting and high rates of surrogate decision-making for this population,^15,17,18,19,20,21,22,23,24,25,26,27^ we conducted a study to evaluate concordance between surrogate decision-maker goals of care and the medical treatments actually received by incapacitated older adults. Furthermore, because code status is an important order that guides care in emergent situations, we also examined whether code status orders were consistent with the goal of care.

## Methods

### Study Setting

This prospective cohort study was conducted in medical units and medical intensive care units (ICUs) in 3 tertiary care hospitals in an urban Midwestern area from April 27, 2012, to July 10, 2015. At the time of the study, the participating hospitals did not have formal procedures for documenting treatment preferences. Patients without formal code status orders were assumed by hospital policy to have full code status. The Indiana University institutional review board approved this study. Surrogates provided written informed consent for themselves and the patients to participate. This report follows Strengthening the Reporting of Observational Studies in Epidemiology (STROBE) reporting guidelines for cohort studies.

### Participants

Participants were patient-surrogate dyads. Eligible patients were hospitalized, were 65 years or older, and lacked the capacity to make medical decisions during the index admission. The patient’s primary surrogate was identified by the patient’s primary hospital physician. To be eligible, surrogates had to have considered at least 1 of the following major decisions during the patient’s current hospital stay: whether to withhold or withdraw life-sustaining therapy, whether to allow a procedure or surgery, or to determine appropriate discharge placement for the patient.

### Study Procedures

Potentially eligible patients were identified through medical record review. The research assistant (RA) (E.S.B.) then conducted a brief screening interview with the patient’s primary physician in which the physician was asked (1) if the patient was unable to make medical decisions and (2) if a surrogate had faced 1 of the 3 categories of decisions. Each patient’s surrogate medical decision-maker was approached for enrollment. A baseline interview was conducted with surrogates within 3 and 10 days of hospital admission by telephone or in person. A follow-up interview was conducted 6 to 8 weeks after discharge. Trained RAs (including E.S.B.) reviewed the medical records to identify treatments received.

### Measures

In baseline interviews, the surrogate provided data about patient and surrogate demographic characteristics. Income was assessed by asking surrogates whether they were comfortable with their income as opposed to being assessed by their actual income owing to high nonresponse rates associated with the latter. At the baseline and follow-up interviews, surrogates were asked to identify the best goal of care for the patient, in the surrogate’s judgment, from the following options: comfort, intermediate care, or life-sustaining treatment. The question probed “If you had to make a choice at this time, do you think the best course of treatment for (the patient) would be: (1) focused on relieving pain and discomfort as much as possible and forgoing measures to prolong life (comfort care); (2) in between, where there may be some care in the hospital but you might refuse care that would be too burdensome (intermediate); or (3) focused on extending life as much as possible, even if it means having more pain and discomfort from treatments (life-sustaining).” Communication quality was measured using the Family Inpatient Communication Survey (FICS-30), a validated survey that shows high reliability in measuring communication experiences for hospital surrogates.^28^ The FICS-30 scores can range from 30 to 150, with a higher score denoting better communication quality.

The investigators created a list of common hospital interventions that were assessed for discordance with each goal of care based on a review of the literature^12,19,24,29^ and consensus of the research team. Because of its importance in guiding emergency care, code status was also compared with each goal of care for concordance. Receipt of medical interventions was abstracted from the patients’ electronic medical records by a trained RA for index hospitalizations.

Because goals of care may change during the hospitalization, the follow-up interview reminded the surrogate of the goal they had selected at the baseline interview by asking whether the goal of care changed during the hospital stay. If a change in goals occurred, the notes in the medical record were reviewed by a trained RA to determine whether care provided was concordant with the goals of care active at the time. This process relied on notes in the medical record from physicians, social workers, and other clinicians documenting conversations concerning goals of care. If the RA was uncertain whether the care received was concordant or discordant, the research team discussed the case and made a final determination.

### Statistical Analysis

Data were analyzed from October 5, 2018, to December 5, 2019. A dichotomous variable was created to reflect whether the patient had 1 or more instances of discordant care. We then identified variables that we hypothesized a priori may be associated with discordant care based on clinical experience and the literature: patient and surrogate age and sex, patient race, marital status, living arrangement, and whether the patient had a living will. Surrogate factors included comfort with income, communication, religion, relationship to the patient, and preference for making decisions. We first conducted bivariate analyses to determine which variables were significantly associated with discordant care using 2-sample 2-tailed *t* tests for continuous variables and Pearson χ^2^ tests for categorical variables. The results of these analyses were used to inform a multivariable analysis, using logistic regression models on the dichotomous outcome. The multivariable analysis chose the included variables through the significance of the bivariate results and the clinical relevance of those a priori chosen variables. These bivariate analyses also allowed us to determine whether and how categorical variables should be collapsed into fewer categories to ensure consistent and appropriate specification in the multivariable model. All analytic assumptions were verified. The odds ratio (OR) for surrogate communication (FICS-30) was provided per 10-point change in score. Two-sided Fisher exact test was used instead of the Pearson χ^2^ test when more than 20% of cells had expected counts of less than 5. All analyses were performed using SAS, version 9.4 (SAS Institute, Inc).

The variables chosen a priori for the model were surrogate age, sex, race, income, educational attainment, and relationship to the patient. Additional surrogate variables included in the model based on 2-sided *P* ≤ .20 (ie, in bivariate analysis with concordance) were religion, prior experience caring for a family member, rating of communication quality, and preference for control of medical decisions.

Because consensus regarding whether some treatments could be regarded as palliative in nature may be lacking, we conducted a sensitivity analysis using a more narrow definition of discordant care, in which treatment that was discordant with comfort care included any of the following: ICU admission, intubation or ventilation, surgery, or receiving cardiopulmonary resuscitation (CPR).

## Results

Contact was attempted with 799 surrogates (Figure). A total of 470 patient-surrogate dyads could be reached to confirm inclusion criteria, 369 dyads were enrolled (78.5% enrollment rate), and 363 dyads were included in the final cohort. Among patients, 223 (61.4%) were women and 140 were men (38.6%), with a mean (SD) age of 81.8 (8.3) years. Among surrogates, 257 were women (70.8%) and 106 were men (29.2%), with a mean (SD) age of 58.3 (11.2) years (Table 1). Five surrogates withdrew and 1 was excluded for declining to answer the course of treatment survey items at either point.

**Figure.**
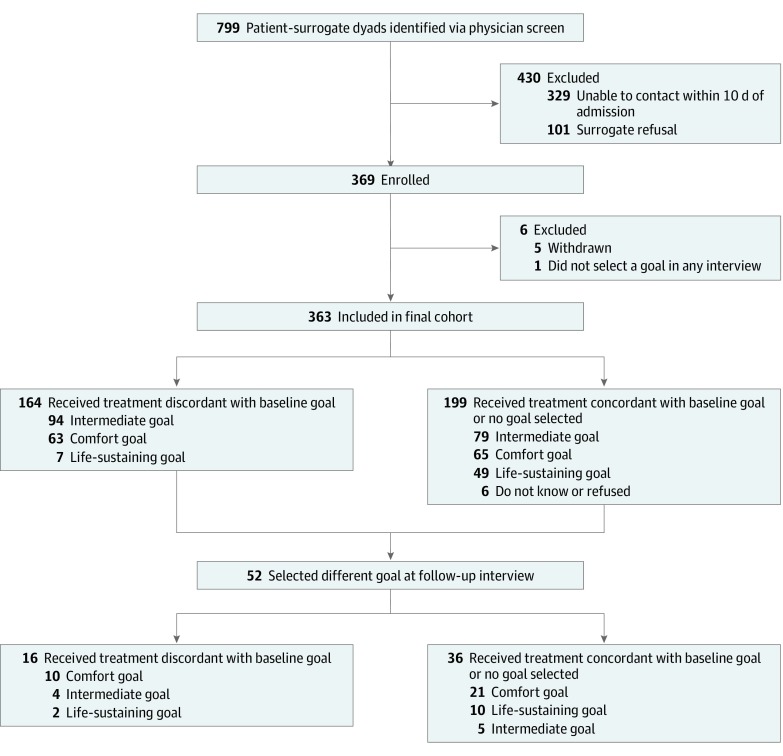
Participant Flow

**Table 1.** Characteristics of Participating Patients and Surrogates

Characteristic	Dyad Participants^a^	
	Patient (n = 363)	Surrogate (n = 363)
Age, mean (SD)	81.8 (8.3)	58.3 (11.2)
Female	223 (61.4)	257 (70.8)
Race		
Black	101 (27.8)	103 (28.5)
White	250 (68.9)	249 (68.8)
Other	12 (3.3)	10 (2.8)
Hispanic	3 (0.8)	3 (0.8)
Comfort level with income		
Comfortable	NA	202 (56.4)
Just enough to make ends meet	NA	116 (32.4)
Not enough to make ends meet	NA	40 (11.2)
Educational level, y		
<12	NA	22 (6.1)
12	NA	123 (34.1)
13-16	NA	172 (47.7)
≥17	NA	44 (12.2)
Religion		
None	22 (6.1)	17 (4.7)
Protestant	287 (79.9)	291 (80.2)
Catholic	41 (11.4)	38 (10.5)
Other	9 (2.5)	17 (4.7)
Marital status		
Married	116 (32.0)	239 (65.8)
Single	15 (4.1)	52 (14.3)
Divorced	53 (14.6)	59 (16.3)
Widowed	174 (47.9)	9 (2.5)
Living with a partner	5 (1.4)	4 (1.1)
Relationship to patient		
Spouse	NA	62 (17.1)
Son	NA	71 (19.6)
Daughter	NA	17 (47.1)
Other	NA	59 (16.3)

Abbreviation: NA, not applicable.

^a^ Totals for some variables do not sum to 363 owing to missing data. Unless otherwise indicated, data are expressed as number (percentage) of participants.

At the baseline interview, 128 of 369 enrolled participants (34.7%) selected the comfort-focused goal. The follow-up interview was completed with 328 of the enrolled surrogates. At the follow-up interview, 52 surrogates indicated that their goal of care had changed at some point during the hospitalization (Table 2). In these cases, RAs reviewed notes in the medical record to determine whether care had been consistent with the goal active at the time of each treatment.

**Table 2.** Discordant Treatment Received by Patients During Hospitalization, by Goal of Care Selected at Baseline or Follow-up

Goal or treatment	Baseline (n = 363)	Follow-up for those whose goals changed from baseline (n = 52)^a^
Selected goal of comfort care, No.	128	31
Received any treatments discordant with comfort care, No. (%)^b^	63 (49.2)	10 (32.3)
Chemotherapy	0	0
ICU (no PCU)	22 (17.2)	6 (19.4)
Intubation/ventilation (except palliative) (n = 77)	18 (14.1)	4 (12.9)
Artificial nutrition	23 (18.0)	6 (19.4)
Surgery (except palliative)	13 (10.2)	1 (3.2)
Procedure (except palliative)	38 (29.7)	6 (19.4)
Dialysis/CVVH	3 (2.3)	1 (3.2)
Resuscitation	1 (0.8)	0
Full code	41 (32.0)	6 (19.4)
Blood transfusion	16 (12.5)	3 (9.7)
Selected goal of intermediate care, total No.	173	9
Any treatments discordant with intermediate care, No (%)^b^	94 (54.3)	4 (44.4)
Resuscitation	9 (5.2)	0
Full code	93 (53.8)	4 (44.4)
Selected goal of life-sustaining interventions, total No.	56	12
Any treatments discordant with life sustaining interventions, No (%)^b^	7 (12.5)	2 (16.7)
Hospice	0	0
DNR	7 (12.5)	2 (16.7)
Indicated don’t know for goal	6	0
Total who had treatment discordant with goal by time point,^c^ No (%)	164 (45.2)	16 (30.8)

Abbreviations: CVVH, continuous venovenous hemofiltration; DNR, do not resuscitate; ICU, intensive care unit; PCU, progressive care unit.

^a^ Fifty-two patients had goals that changed between the baseline and follow-up interviews. Of 363 patients, 169 (46.6%) had 1 or more discordant treatments at baseline or follow-up (164 had treatments discordant with the baseline goal, 11 had discordant treatments at both points, and 5 had discordant treatments only at follow-up).

^b^ The sum of specific treatments exceeds the number with any treatment discordant with each goal, because participants may have experienced more than 1 discordant treatment.

^c^ The sum of patients with discordance at each point exceeds the total of 169, because 11 patients had discordance at 2 points.

We found that 169 patients (46.6%) received at least 1 medical treatment discordant with their surrogate’s identified goals of medical care. At baseline, 63 patients had 1 or more treatments discordant with a comfort-focused goal; 94, discordant with an intermediate goal; and 11, discordant with aggressive life-sustaining goal (Figure). The most common reason for discordant medical treatment involved patients having full code status although their surrogate identified either a treatment plan with comfort measures only (41 cases) or an intermediate treatment plan (93 cases) (Table 2). Among patients whose surrogates identified a preference for comfort measures only or an intermediate goal of treatment, 10 received CPR.

In the bivariate analysis, several patient and surrogate factors were associated with discordance (Table 3). In the multivariable model (Table 4), factors associated with lower odds of discordant treatment included communication quality (adjusted OR [AOR] per 10-point increase on the FICS-30 survey, 0.91; 95% CI, 0.60-0.95), patient living in a facility (AOR, 0.44; 95% CI, 0.23-0.82), and more frequent surrogate visitation with the patient (AOR, 0.43; 95% CI, 0.23-0.82). Surrogate marital status of being single (AOR, 1.92; 95% CI, 1.01-3.66), more than 1 family member being involved in the decision-making process (AOR, 1.84; 95% CI, 1.05-3.21), and having no religious affiliation (AOR, 4.87; 95% CI, 1.12-21.09) were each associated with higher odds of discordance.

**Table 3.** Patient and Surrogate Factors Associated With Discordance With Goals of Care: Bivariate Analysis Initial and Broad Definitions

Factor	Definition of discordance^a^					
	Broad (n = 363)			Narrow (n = 363)		
	Discordant care (n = 169)	Concordant care (n = 194)	*P* value^b^	Discordant care (n = 62)	Concordant care (n = 301)	*P* value^b^
**Patient **						
Age, mean (SD)	79.5 (7.7)	83.9 (8.3)	<.001	80.1 (8.2)	82.2 (8.3)	.08
Female (vs male)	101 (45.3)	122 (54.7)	.54	39 (17.5)	184 (82.5)	.79
Black race (vs other)	59 (58.4)	42 (41.6)	.02	18 (17.8)	83 (82.2)	.97
Marital status single/etc (vs with a partner)	106 (43.8)	136 (56.2)	.14	40 (16.5)	202 (83.5)	.69
Living arrangement institution (vs private)	38 (37.6)	63 (62.4)	.03	12 (11.9)	89 (88.1)	.10
Visitation with patient more than weekly/lives with surrogate (vs weekly or less frequently)	114 (43.2)	150 (56.8)	.02^b^	38 (14.4)	226 (85.6)	.01^b^
Patient discussed medical wishes with anyone (vs no)	126 (47.5)	139 (52.5)	.43	51 (19.2)	214 (80.8)	.08
Patient has living will (vs no)	68 (38.9)	107 (61.1)	.01	26 (14.9)	149 (85.1)	.37
**Surrogate **						
Age, mean (SD)	56.4 (12.6)	59.9 (9.7)	.005	58.2 (11.3)	58.3 (11.3)	.94
Female	123 (47.9)	134 (52.1)	.44	43 (16.7)	214 (83.3)	.78
Race						
Black	60 (58.3)	43 (41.7)	.01	19 (18.4)	84 (81.6)	.81
White	105 (42.2)	144 (57.8)		41 (16.5)	208 (83.5)	
Other	3 (30.0)	7 (70.0)		1 (10.0)	9 (90.0)	
Hispanic	2 (66.7)	1 (33.3)	.44	1 (33.3)	2 (66.7)	.14
Comfort level with income						
Comfortable	88 (43.6)	114 (56.4)	.23	43 (21.3)	159 (78.7)	.04
Just enough to make ends meet	62 (53.4)	54 (46.6)		15 (12.9)	101 (87.1)	
Not enough to make ends meet	18 (45.0)	22 (55.0)		3 (7.5)	37 (92.5)	
Educational level, y						
<12	12 (54.5)	10 (45.5)	.68	1 (4.5)	21 (95.5)	.23
12	53 (43.1)	70 (56.9)		25 (20.3)	98 (79.7)	
13-16	84 (48.8)	88 (51.2)		31 (18.0)	141 (82.0)	
≥17	20 (45.5)	24 (54.5)		5 (11.4)	39 (88.6)	
Religious affiliation						
None	14 (82.4)	3 (17.6)	.01	4 (23.5)	13 (76.5)	.71
Protestant	134 (46.0)	157 (54.0)		48 (16.5)	243 (83.5)	
Catholic	16 (42.1)	22 (57.9)		8 (21.1)	30 (78.9)	
Other	5 (29.4)	12 (70.6)		2 (11.8)	15 (88.2)	
Marital status						
Married	103 (43.1)	136 (56.9)	.04	45 (18.8)	194 (81.2)	.61
Single	30 (57.7)	22 (42.3)		9 (17.3)	43 (82.7)	
Divorced	33 (55.9)	26 (44.1)		8 (13.6)	51 (86.4)	
Widowed	3 (33.3)	6 (66.7)		0	9 (100)	
Opposite-sex partner	0	4 (100)		0	4 (100)	
Same-sex partner	0	0		0	0	
Relationship to patient						
Spouse	34 (54.8)	28 (45.2)	.34	11 (17.7)	51 (82.3)	.81
Son	28 (39.4)	43 (60.6)		11 (15.5)	60 (84.5)	
Daughter	81 (47.4)	90 (52.6)		32 (18.7)	139 (81.3)	
Other	26 (44.1)	33 (55.9)		8 (13.6)	51 (86.4)	
REALM score >6th grade literacy^c^	61 (48.4)	65 (51.6)	.61	22 (17.5)	104 (82.5)	.89
Prior experience caring for a hospitalized family member	143 (44.3)	180 (55.7)	.01	55 (17.0)	268 (83.0)	.94
Patient illness unexpected at the time of hospitalization	133 (48.5)	141 (51.5)	.37	55 (20.1)	219 (79.9)	.02^b^
≥2 family members involved in the decision-making process (vs 0-1)	95 (51.4)	90 (48.6)	.07	35 (18.9)	150 (81.1)	.26
Surrogate/relationship conflict existed	62 (50.8)	60 (49.2)	.25	16 (13.1)	106 (86.9)	.15
FICS-30 total score, median (IQR)^d^	120 (109-135)	127 (116-143)	<.001	127 (112-141)	124 (113-139)	.58
Surrogate preference for making medical decisions						
Prefers to make the final selection	8 (53.3)	7 (46.7)	.18	4 (26.7)	11 (73.3)	.33
Prefers to make the final selection after seriously considering physician’s opinion	62 (43.4)	81 (56.6)		25 (17.5)	118 (82.5)	
Prefers to share decision-making with physician	87 (52.1)	80 (47.9)		26 (15.6)	141 (84.4)	
Prefers physician makes the final decision about medical treatments with own opinion considered	9 (32.1)	19 (67.9)		7 (25.0)	21 (75.0)	
Prefers that all treatment decisions be made by physician	3 (30.0)	7 (70.0)		0	10 (100)	

Abbreviations: FICS-30, Family Inpatient Communication Survey; IQR, interquartile range; REALM, Rapid Estimate of Adult Literacy in Medicine.

^a^ Data are expressed as number (percentage) of participants by row unless otherwise indicated. Narrow definition of treatment discordant with comfort care includes intensive care unit admission, intubation or ventilation, surgery, or receiving cardiopulmonary resuscitation.

^b^ Calculated from χ^2^ tests for categorical variables, two sample two-tailed *t* tests for linear continuous variables given with mean (SD), and Wilcoxon nonparametric tests for nonlinear continuous variables given with median (IQR).

^c^ Scores range from 0 to 66, with higher scores indicating the patient will be able to read most patient education materials.

^d^ Scores range from 30 to 150, with higher scores indicating better communication quality.

**Table 4.** Patient and Surrogate Factors Associated With Discordance With Goals of Care by Multivariable Logistic Regression

Factor	Broad definition (n = 363)		Narrow definition (n = 363)	
	AOR (95% CI)	*P* value^a^	AOR (95% CI)	*P* value^a^
**Patient Factors**				
Age	0.96 (0.92-1.00)	.07	0.97 (0.92-1.03)	.28
Female (vs male)	1.38 (0.72-2.64)	.34	1.80 (0.77-4.18)	.18
Black race (vs other)	1.75 (0.91-3.34)	.09	1.26 (0.55-2.86)	.58
Marital status single/etc (vs partnered)	0.47 (0.21-1.07)	.07	0.39 (0.15-1.02)	.06
Institutional living arrangement (vs private)	0.44 (0.23-0.86)	.02	0.37 (0.16-0.87)	.02
Visitation with surrogate more than weekly/lives with surrogate (vs weekly or less frequently)	0.43 (0.23-0.82)	.01	0.37 (0.17-0.81)	.01
Discussed medical wishes with anyone (vs no)	1.73 (0.90-3.31)	.10	2.19 (0.91-5.27)	.08
Has living will (vs no)	0.85 (0.46-1.55)	.59	0.76 (0.37-1.59)	.47
**Surrogate Factors**				
Age	0.97 (0.94-1.01)	.14	1.01 (0.97-1.06)	.53
Female (vs male)	1.05 (0.33-3.37)	.94	0.84 (0.20-3.47)	.81
Comfort level with income				
Comfortable (vs not enough)	1.86 (0.75-4.64)	.39	5.75 (1.15-28.82)	.01
Just enough to make ends meet (vs not enough)	2.02 (0.78-5.24)	.24	2.71 (0.51-14.45)	.81
Educational level ≤12 y (vs >12 y)	0.80 (0.44-1.44)	.45	1.35 (0.66-2.78)	.41
Marital status single/etc (vs partnered)	1.92 (1.01-3.66)	.047	0.84 (0.37-1.88)	.66
Relationship to patient (vs spouse)				
Son	0.49 (0.10-2.48)	.78	2.29 (0.37-14.29)	.68
Daughter	0.55 (0.14-2.19)	.92	3.56 (0.62-20.45)	.14
Other	0.38 (0.09-1.59)	.23	1.34 (0.23-7.83)	.49
REALM score >6th grade	0.63 (0.36-1.12)	.11	0.82 (0.41-1.64)	.57
Prior experience caring for a hospitalized family member (vs No)	0.48 (0.19-1.23)	.12	1.29 (0.43-3.90)	.65
≥2 family members involved in the decision-making process (vs 0-1)	1.84 (1.05-3.21)	.03	1.44 (0.72-2.88)	.30
FICS-30 total score^b^	0.81 (0.69-0.95)	.01	1.05 (0.86-1.29)	.62
Surrogate preference for making medical decisions (vs shared)				
Surrogate	0.87 (0.50-1.51)	.97	1.02 (0.52-2.01)	.79
Physician	0.74 (0.27-2.00)	.64	1.29 (0.38-4.46)	.68
No religious affiliation (vs others)	4.87 (1.12-21.09)	.03	1.76 (0.45-6.85)	.41
Surrogate and physician disagreed about the patient’s prognosis (vs agreed)	0.83 (0.37-1.90)	.67	1.59 (0.63-3.98)	.33

Abbreviations: AOR, adjusted odds ratio; FICS-30, Family Inpatient Communication Survey; REALM, Rapid Estimate of Adult Literacy in Medicine.

^a^ Calculated from multivariable logistic regression models.

^b^ Indicates per 10-point change in FICS-30 score.

Under the narrow definition of discordance, 62 patients (17.1%) had discordant care. Only living in a facility (AOR, 0.37; 95% CI, 0.16-0.87), surrogate visitation more than weekly (AOR, 0.37; 95% CI, 0.17-0.81), and the surrogate describing their income as comfortable (AOR, 5.75; 95% CI, 1.15-28.82) were significantly associated with discordance (Table 4).

## Discussion

Almost half of the patients in this study had at least 1 medical treatment or code status order that was discordant with the goal of care identified by their surrogates. The most common source of discordance was having a full code status when the surrogate’s preferred goal was comfort measures only or an intermediate goal. Given that the default order for code status is full code, there may be many cases where needed discussions with the surrogate do not occur or result in an order change. This finding is distinguishable from a recent study conducted in nursing homes where 98% of patients whose surrogates preferred comfort care had a directive for DNR^30^ but similar to a recent study in which many patients or surrogates who highly valued comfort had orders for CPR.^31^ The longer time frame of nursing facility admissions may allow for higher-quality discussions, or facilities may have a more standard approach to addressing code status uniformly. The present study found there may be serious consequences to failing to document preferences for comfort care. For example, 10 patients in this study had surrogates who did not select a goal of life-sustaining treatment but who received CPR. Overall, using a narrower definition of discordance that focused on resuscitation, ICU care, and surgery, nearly 1 in 5 patients still had discordant treatments.

The results of this study suggest that implementation of DNR orders for patients whose surrogates have identified that they do not want aggressive medical interventions is an important part of ensuring concordant medical treatment. Prior work^32,33^ has identified barriers to writing DNR orders in a timely fashion. Studies have found that code status is typically addressed only when a patient faces poor prognosis or poor quality of life rather than on a routine basis. Perhaps as a consequence, code status orders are often discordant with patient or family preferences, and surrogates are often unable to correctly identify a patient’s current code status during hospitalization.^32,33^ A major challenge with the execution of appropriately timed DNR orders is the surrogate’s fear that the patient will no longer receive other medical care that the surrogate believes is still appropriate.^29,34,35,36,37,38,39,40,41^ Ensuring that code status is not conflated with a rejection of other interventions is important to documenting code status appropriately. In addition, physician communication quality is poor when having conversations about code status and when surrogates are involved in making code status order decisions, and code status orders are entered later and closer to the time of the patient’s death.^42,43^

This study found several patient and surrogate factors associated with discordant care. Patients who lived in a nursing facility were more likely to experience care that was concordant with the surrogate’s stated goals of care. Conversely, we also found that the physical proximity of the surrogate to the patient was important, with less discordance occurring for patients who either lived with a surrogate or saw them more than weekly. These findings may seem contradictory; however, patients who live in a facility often have advanced frailty or serious illness, and there may be greater consensus about limiting life-sustaining treatments in this population.^44,45^ More frequent contact with the patient may help the surrogate understand the patient’s wishes or medical condition and may make decision-making easier. Surrogates who live with or see the patient more often may also be stronger advocates for the patient. Finally, higher communication quality with clinical staff, as rated by the surrogate, was associated with lower discordance, signaling that early communication may lead to earlier decision-making and better documentation of decisions.^36,46,47^

There were many instances of patients receiving life-sustaining medical interventions despite their surrogates identifying that some limitations in treatment were preferred, including 10 patients who received CPR although their goal of care was comfort or intermediate care. Surrogates’ overall preferences expressed during study interviews may differ from specific treatments for several reasons, including the specific context of the treatment decision, framing by the physician, or lack of communication about the relationship of the treatment to broader goals.^31^ In the hospital, one way to address this kind of discordance may be to involve palliative care when a patient or surrogate expresses that the goal of medical care is comfort.^48,49^ Palliative care physicians are trained to provide support for the patient and family to ensure that patients who wish to forgo aggressive medical interventions are provided pain control, symptom management, and psychosocial, spiritual, and/or bereavement support.^48^

### Limitations

This study has several limitations. First, this study did not address the reasons behind the surrogate’s goals of care choice for the patient. Therefore, this study can only report discordance in medical treatments and goals of care, not reasons why the discordance is occurring. Reasons could include difficulty communicating preferences to the medical team or a lack of shared understanding about how the goals translate into specific medical treatments. Second, this study did not address concordance between the surrogate’s and patient’s goals of care; however, this was not possible because the patients enrolled in this study all lacked capacity and thus were unable to make their own medical decisions. Third, this study did not address whether surrogates perceived discordance in care but rather whether there was actual discordance in medical care received. Also, this study did not explore physician preferences for the patient’s treatment. In addition, this study measured discordance between surrogate-identified goals of care and treatments the patient received based on a definition developed by the study team, because no standardized definition or method of determining discordance is available. Our research team made judgments about the treatments that were discordant with certain goals that others may disagree with. A further challenge is that goals changed during hospitalization for some patients. Because goals of care are not recorded in the electronic medical record in a standardized manner, we relied on medical record review and judgments by the research team to determine from the electronic medical record when goals changed. Establishing a standardized definition and understanding of discordance with treatment goals is an important direction for future research. To address the limitation of not having a universally excepted standard of discordance, the study team also created a narrow definition of discordance that included only ICU care and major surgery.

## Conclusions

This study found that nearly half of patients received at least 1 medical treatment or code status order that was discordant with their surrogate’s identified goals of care, resulting in patients receiving potentially unwanted aggressive medical treatments, including CPR, surgery, and intubation. Future work is needed to understand the surrogates’ perspective on the concordance between goals of care and treatments. The finding that communication quality was associated with lower discordance suggests that improving communication between surrogates and clinicians may help. Strategies should proactively identify the surrogate’s goal of care and discuss how specific treatments may or may not help achieve that goal.
